# Clinical features of nivolumab-induced Stevens-Johnson syndrome/toxic epidermal necrolysis: retrospective analysis based on case reports

**DOI:** 10.3389/fimmu.2025.1563100

**Published:** 2025-03-18

**Authors:** Ronghui Li, Haibo Lei, Chunjiang Wang, Xiang Liu

**Affiliations:** ^1^ Department of Clinical Pharmacy, Xiangtan Central Hospital (The Affiliated Hospital of Hunan University), Xiangtan, Hunan, China; ^2^ Department of Pharmacy, The Third Xiangya Hospital, Central South University, Changsha, Hunan, China

**Keywords:** nivolumab, toxic epidermal necrolysis, Stevens-Johnson syndrome, cutaneous adverse reactions, immune-related adverse events

## Abstract

**Background:**

Stevens – Johnson syndrome/toxic epidermal necrolysis (SJS/TEN) is a life-threatening adverse reaction to nivolumab. This study investigated the clinical features of nivolumab induced SJS/TEN to provide evidence for diagnosis and treatment.

**Methods:**

Relevant articles on nivolumab induced SJS/TEN published before December 31, 2024 were collected by searching the database, and then extracting the data for summary analysis.

**Results:**

Thirty-one patients were enrolled with a median age of 65 years (range 43, 86). SJS/TEN appear at a median of 5.5 weeks (range, 0.9 108). Bullae/blisters (64.5%), erythema (54.8%), skin rash (54.8%), epidermal detachment (29.0%) and pain (29.0%) were the main skin symptoms. Skin biopsy showed epidermal necrosis (41.9%), keratinocytic necrosis (38.7%), interface dermatitis (29.0%) and inflammatory cell infiltration (45.2%). After stopping nivolumab and receiving treatment, 74.2% of the patients had improvement in skin symptoms, and 22.6% of the patients died of TEN.

**Conclusion:**

As a rare immune-related adverse event of nivolumab, SJS/TEN should be closely monitored during the treatment. Nivolumab induced SJS/TEN has a long incubation period, serious clinical symptoms and poor prognosis.

## Introduction

Nivolumab, an immune checkpoint inhibitor (ICIs) approved in 2014, is used to treat a variety of malignancies such as melanoma, renal cell carcinoma, head and neck squamous cell carcinoma, and non-Hodgkin’s lymphoma (1). Nivolumab has demonstrated remarkable anti-tumor activity; however, it has also been associated with a significant incidence of immune-related adverse events (irAEs). Nivolumab may trigger multi-system irAEs, such as skin, gastrointestinal tract, endocrine system, hepatotoxicity, kidney and lung toxicity (2). Among them, the incidence of cutaneous immune−related adverse events (cirAEs) caused by nivolumab is the highest, which greatly affects patients’ quality of life and anti-tumor treatment decisions. Pruritus (30.1%), maculopapular rash (24.7%) and vitiligo (16.1%) were more common (3). Stevens – Johnson syndrome/toxic epidermal necrolysis (SJS/TEN) is a life-threatening skin-mucous reaction characterized by vesicles and generalized epidermolysis, which may be associated with multiple system involvement (4). SJS/TEN is a rare irAEs of nivolumab. Unfortunately, knowledge about nivolumab-induced SJS/TEN comes mainly from case reports. The clinical regularity of nivolumab induced SJS/TEN still needs to be explored. This study summarized the clinical manifestations, treatment and prognosis of nivolumab induced SJS/TEN, and provided a reference for clinicians to diagnose and treat nivolumab induced SJS/TEN.

## Methods

### Retrieval method

Case reports and case series articles on nivolumab induced SJS/TEN were included by searching the database. Databases involved in this study include PubMed, EMbase, Web of Science, Cochrane Library, WanFang Data, China National Knowledge Infrastructure, China Science and Technology Journal Database, China Biology Medicine disc. The search language is limited to Chinese and English, and the search time ends on December 31, 2024. The retrieval method is the combination of the subject heading and text word terms through the Boolean operator connection. The search regimens included “nivolumab” OR “immune checkpoint inhibitor” OR “PD1 inhibitor” AND “Stevens-Johnson syndrome/toxic epidermal necrolysis” OR “Stevens-Johnson syndrome” OR “toxic epidermal necrolysis” OR “SJS/TEN” OR “immune-related adverse events” OR “cutaneous immune−related adverse events”.

### Inclusion and exclusion criteria

Case reports and case series of nivolumab induced SJS/TEN were included. Duplicate articles, reviews, mechanism studies, and non-nivolumab induced SJS/TEN were excluded.

### Clinical data extraction

The author designed the table and extracted the patient’s clinical data, including country, gender, age, incubation period, indications, disease history, concomitant medications, clinical manifestations, other autoimmune related adverse events, Nikolsky sign, SJS/TEN type, other autoimmune related adverse events, skin biopsy, treatment and outcome.

### SJS/TEN type

SJS is defined as the epidermolysis area < 10% body surface area, TEN is defined as the epidermolysis area > 30% body surface area, SJS/TEN overlap is defined as the epidermolysis area of 10% ~ 30% body surface area (5).

### Statistical analysis

SPSS 26.0 software was used for statistical analysis of the extracted data. The counting data is represented by n (%), and the measurement data is the median value (range minimum, maximum).

## Results

### Basic characteristics

**Figure 1:**
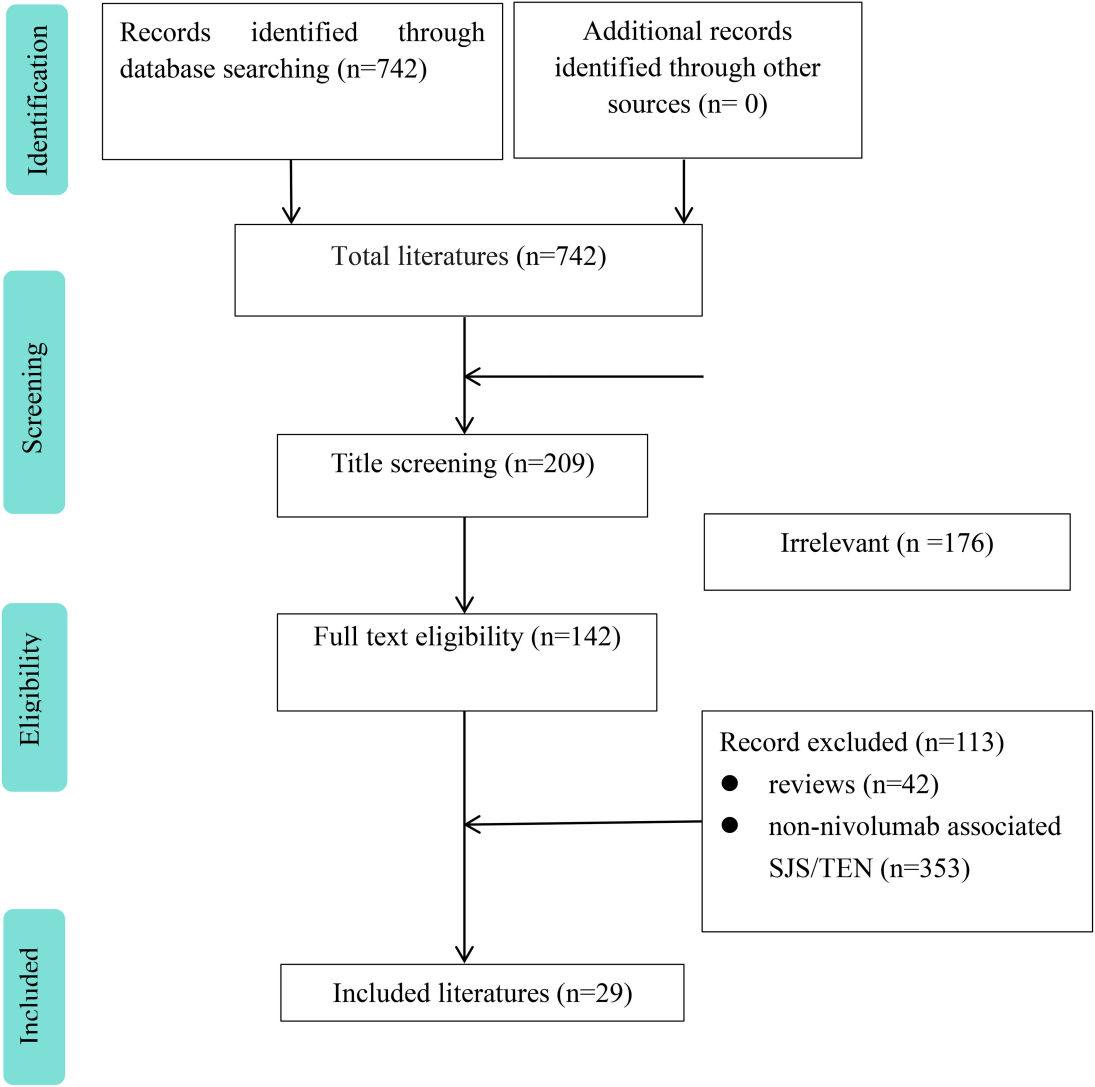
Flow chart of study selection process for reported cases of nivolumab-induced SJS/TEN.

After screening, 29 studies were eventually included, including 31 patients (**Figure 1**). **Table 1** summarizes the basic information of 31 patients. The median age of the 31 patients was 65 years (range 43, 86), including 18 males (58.1%) and 13 (41.9%) females. The top three reporting countries were the USA (11 cases, 35.5%), Japan (7 cases, 22.6%) and the United Kingdom (5 cases, 16.1%). The main cancer type in these patients was melanoma (9 cases, 29.0%) and lung cancer (6 cases, 19.4%). The onset time of SJS/TEN was 5.5 weeks (range 0.9,108) after the initial dose, including 7 weeks (range 0.9,108) for TEN and 6 weeks (range 1, 36) for SJS. The median cycle at the onset of SJS/TEN is 2 cycles (range 1, 77). Fifteen patients reported duration of onset of SJS/TEN after discontinuation, with a median duration of 10 days (range 2, 180). Sixteen (51.6%) patients had a history of diseases, including atrial fibrillation, hypertension, liver cirrhosis, hyperlipidemia, chronic kidney disease, follicular lymphoma, diabetes mellitus, hypersplenism, hepatitis C infection, Parkinson disease, peripheral neuropathy, hypothyroidism, hepatocellular carcinoma, gastro-esophageal reflux disease, osteoporosis. Sixteen patients were treated with other drugs, of which 7 (22.6%) received nivolumab and ipilimumab.

**Table 1:** Basic data of 31 patients with nivolumab-induced SJS/TEN.

Parameter	Classification	Value
Sex	male	18 (58.1%)
	female	13 (41.9%)
Age	year	65 (43, 86) ^b^
Country	USA	11 (35.5%)
	Japan	7 (22.6%)
	United Kingdom	5 (16.1%)
	Spain	2 (6.5%)
	France	1 (41.9%)
	Brasil	1 (3.2%)
	China	1 (3.2%)
	India	1 (3.2%)
	Italy	1 (3.2%)
	Australia	1 (3.2%)
Onset time	cycle (25)^a^	2 (1, 77) ^b^
Onset time after initial administration (14)^a^	weeks	5.5 (0.9, 108) ^b^
TEN (7)^a^	weeks	7 (0.9, 108) ^b^
SJS (6)^a^	weeks	6 (1, 36) ^b^
Onset time since last dosing (15)^a^	days	10 (2, 180) ^b^
Indication	melanoma	9 (29.0%)
	lung cancer	6 (19.4%)
	squamous cell carcinoma	3 (9.7%)
	gastric cancer	3 (9.7%)
	esophageal cancer	2 (6.5%)
	hepatocellular carcinoma	2 (6.5%)
	squamous cell vulvar carcinoma	1 (3.2%)
	colon adenocarcinoma	1 (3.2%)
	recurrent follicular lymphoma	1 (3.2%)
	esophageal cancer	1 (3.2%)
	epithelioid pleural mesothelioma	1 (3.2%)
	renal Cell Carcinoma	1 (3.2%)
Medical history	atrial fibrillation, hypertension, liver cirrhosis, hyperlipidemia, chronic kidney disease, follicular lymphoma, diabetes mellitus, hypersplenism, hepatitis C infection, Parkinson disease, peripheral neuropathy, hypothyroidism, hepatocellular carcinoma, gastro-esophageal reflux disease, osteoporosis	7 (22.6%)
Drug combination (16)^a^	ipilimumab	7 (22.6%)
	oxaliplatin, capecitabine, lorazepam, allopurinol, lactulose, fluconazole, 5-fluorouracil, leucovorin, furosemide, spironolactone, folic acid, docetaxel, gabapentin, oxycodone, metformin, diltiazem, atorvastatin, apixaban, lansoprazole, bromide, omeprazole, pantoprazole, tiotropium carbidopa/levodopa, co-trimoxazole	10 (32.3%)

^a^represents the number of cases describing this parameter out of 31 patients.

b Median (minimum, maximum).

### Clinical manifestation

**Table 2** summarizes the clinical manifestations of 31 patients. The main skin symptoms of the patients were bullae/blisters (20 cases, 64.5%), erythema (17 cases, 54.8%), skin rash (17 cases, 54.8%), epidermal detachment (9 cases, 29.0%) and pain (9 cases, 29.0%). A small number of other patients may present with oral erosions (7 cases, 22.6%), skin erosion (6 cases, 19.4%), pruritus (6 cases, 19.4%), desquamation (6 cases, 19.4%), fever (6 cases, 19.4%). 19.4%) and lip crusting (6 cases, 19.4%) Twenty-five (80.6%) patients experienced mucosal involvement, including oral mucosa (21 patients, 67.7%), conjunctiva (10 patients, 32.3%), and genitals (8 patients, 25.8%).

**Table 2:** Clinical features of 31 patients with nivolumab-induced SJS/TEN.

Parameter	Classification	Value
Clinical manifestation	bullae/blisters	20 (64.5%)
	erythema	17 (54.8%)
	skin rash	17 (54.8%)
	epidermal detachment	9 (29.0%)
	pain	9 (29.0%)
	oral erosions	7 (22.6%)
	skin erosion	6 (19.4%)
	pruritus	6 (19.4%)
	desquamation	6 (19.4%)
	fever	6 (19.4%)
	lip crusting	6 (19.4%)
	purpura	3 (9.7%)
	mucosal involvement	25 (80.6%)
	oral mucosa	21 (67.7%)
	conjunctiva	10 (32.3%)
	genitals	8 (25.8%)
	other symptoms: fatigue, odynophagia, diarrhea, oral sloughing, blurry vision, vomiting	7 (22.6%)
Biopsy (26)^a^	epidermal necrosis	13 (41.9%)
	keratinocytic necrosis	12 (38.7%)
	interface dermatitis	9 (29.0%)
	vacuolar alteration	5 (16.1%)
	subepidermal split	4 (12.9%)
	dyskeratotic cells	4 (12.9%)
	subepidermal blister	3 (9.7%)
	detachment of epidermis	2 (6.5%)
	inflammatory cell infiltration	14 (45.2%)
	lymphocytic infiltrate	7 (22.6%)
	eosinophils infiltrate	2 (6.5%)
	neutrophils infiltrate	1 (3.2%)
	lymphocytes and eosinophils infiltrate	1 (3.2%)
	lymphocytes and neutrophils infiltrate	1 (3.2%)
	not reported	2 (6.5%)
Concurrent other autoimmune related adverse events	hepatitis, thrombocytopenia, thyroid dysfunction, type 1 diabetes mellitus, Hashimoto disease	3 (9.7%)
Nikolsky (15)^a^	positive	14 (45.2%)
	negative	1 (3.2%)
Classification	TEN	13 (41.9%)
	SJS	16 (51.6%)
	SJS/TEN overlap	2 (6.5%)

^a^represents the number of cases describing this parameter out of 31 patients.

Nikolsky sign was reported in 15 patients, of whom 14 (45.2%) were positive. Three patients (9.7%) had other autoimmune related adverse events, including hepatitis, thrombocytopenia, thyroid dysfunction, type 1 diabetes mellitus, Hashimoto disease. According to the classification criteria, 16 (51.6%) patients were defined as SJS, 13 (41.9%) as TEN, and 2 (6.5%) as SJS/TEN overlap.

### Skin biopsy

**Table 2** summarizes the skin biopsy of 31 patients. Skin biopsy was performed in 26 of the 31 patients, and the main findings were epidermal necrosis (13 cases, 41.9%), keratinocytic necrosis (12 cases, 38.7%), interface dermatitis (9 cases, 29.0%) and inflammatory cell infiltration (14 cases, 45.2%). These inflammatory cells were mainly lymphocytes (7 cases, 22.6%), followed by neutrophils (2 cases, 6.5%).

### Treatment and outcome

**Table 3** summarizes the treatment regimens and outcomes of 31 patients. Thirty (96.8%) patients withdrew nivolumab and one (3.2%) patient did not report nivolumab status. Twenty-seven (87.1%) patients received systemic steroid therapy and 10 (32.3%) patients received intravenous immunoglobulins (IVIG). Other treatment regimens included topical steroid (4 cases, 12.9%), cyclosporine (2 cases, 6.5%), infliximab (2 cases, 6.5%), etanercept (1 case, 3.2%), ruxolitinib (1 patient, 6.5%), ruxolitinib (1 case,6.5%). 3.2%) and plasmapheresis (1 case, 3.2%). Among patients treated with nivolumab alone, 20 (64.5%) patients experienced improvement in skin symptoms and 4 (12.9%) patients died from TEN. Of the seven patients treated with nivolumab and ipilimumab, three died of multiple organ failure, two improved symptoms, and one did not describe the outcome. The median time to improvement of skin symptoms was 28 days (range 14, 360).

**Table 3:** Treatment and outcomes of 31 patients with nivolumab-induced SJS/TEN.

Parameter	Classification	Value
Treatment	withdraw	30 (96.8%)
	not available	1 (3.2%)
	systemic steroids	13 (41.2%)
	systemic steroids + topical steroid	3 (9.7%)
	systemic steroids + IVIG	6 (19.4%)
	systemic steroids + IVIG + infliximab	2 (6.5%)
	systemic steroids + IVIG + cyclosporine	1 (3.2%)
	systemic steroids + etanercept	1 (3.2%)
	systemic steroids + ruxolitinib	1 (3.2%)
	IVIG + plasmapheresis	1 (3.2%)
	topical steroid + cyclosporine	1 (3.2%)
Outcome (30)^a^	nivolumab alone	24 (77.4%)
	improvement	20 (64.5%)
	die of TEN	4 (12.9%)
	died from original disease	6 (19.4%)
	nivolumab and ipilimumab	7 (22.6%)
	improvement	3 (9.7%)
	die of TEN	3 (9.7%)
	unspecified	1 (3.2%)
	died from original disease	1 (3.2%)
Recovery time (17)^a^	days	28 (14, 360) ^b^

IVIG, intravenous immunoglobulins; TEN, toxic epidermal necrosis.

^a^represents the number of cases describing this parameter out of 31 patients.

^b^Median (minimum, maximum).

## Discussion

Post-marketing data analysis showed that the average incidence of cirAEs was about 25.1%, with a higher proportion of cirAEs in patients receiving anti-CTLA-4 treatment than anti-PD-1/PD-L1 treatment (6, 7). The incidence of cirAEs was higher when different ICIs were combined (8). The incidence of pruritus in patients treated with PD-1 inhibitors ranges from 13% to 20%, while the incidence of SJS/TEN is lower, but the mortality is higher (9). Compared with other drug-induced SJS/TEN, nivolumab induced SJS/TEN tended to have a longer incubation period, with a median time of 5.5 weeks. The different pharmacokinetics may explain the different onset time of drug-induced SJS/TEN. Nivolumab has a half-life of 25 days, and steady-state plasma concentrations are achieved at 12 weeks with biweekly dosing regimens (10). Other drugs, such as lamotrigine, have a half-life of about 23 to 37 hours, with peak blood concentrations 3 hours after administration. Therefore, the mean time of onset of lamotrigine-induced SJS/TEN was 2.8 weeks (11). An alternative explanation is that PD-1 inhibition activates T cells directed against self-antigens, leading to progressive loss of peripheral tolerance in the skin and later development of SJS/TEN (12). SJS/TEN may even occur up to 6 months after withdrawal of nivolumab (13). This is a delayed immune-related event (14). Nivolumab has an approximately 85% occupancy rate on circulating T cells for 85 days and is dose-independent (15).

The ratio of nivolumab induced SJS/TEN was about 1.4 to 1, and the incidence was higher in males than in females. There was no significant sex difference in classical drug-induced SJS/TEN (16). Considering the limited number of included cases, whether gender is related to the occurrence of SJS/TEN needs to be further verified. In addition, patients often have multiple underlying diseases that require a combination of multiple drugs. These drugs are related to SJS/TEN, such as proton pump inhibitors and allopurinol (17). However, based on the time correlation and the improvement of patients’ symptoms after discontinuation of nivolumab, the cause can be identified as nivolumab. However, whether the combination of multiple drugs increases the incidence of SJS/TEN needs further investigation.

Our findings suggest that nivolumab induced SJS/TEN is clinically distinct from that classical drug-induced SJS/TEN. Drug-induced SJS/TEN develops rashes in the first few days of rapid progression, whereas ICI-related skin outbreaks occur weeks to months earlier (18). In our study, about 55% of the cases had a rash prior to blistering and skin sloughing. Nivolumab-induced SJS/TEN is similar to classical drug-induced SJS/TEN in more than 80% of patients with mucosal involvement. Different mucosal involvement of SJS/TEN induced by nivolumab was significantly lower than that of classical SJS/TEN. However, the involvement of different mucous membranes varies, 90% of classical SJS/TEN involve the oral mucosa, 84% involve the eyes, and 60-70% involve the genitals (19, 20). Another note is that there may be a lack of mucosal involvement in patients with extensive epidermal detachment. These may be a unique feature of nivolumab induced SJS/TEN (21). Drug-induced SJS/TEN histology showed epidermal necrosis, keratinocyte apoptosis, basal vacuolar change, subepidermal bullae, subepidermal clefting, and Inflammatory cell infiltration. 1 These inflammatory cells were mainly infiltrated by lymphocytes, followed by eosinophils and neutrophils. Skin biopsies of nivolumab induced SJS/TEN were similar to classical ones. However, nivolumab induced SJS/TEN was found to have less inflammatory cell infiltration than classical SJS/TEN. Wetter et al. reported that 85% of patients had inflammatory cell infiltration, while we found that only about 54% of patients had inflammatory cell infiltration (16). Nivolumab-induced SJS/TEN needs to be distinguished from other skin diseases, such as bullous lichenoid dermatitis, bullous radiation recall dermatitis, bullous pemphigoid, and paraneoplastic pemphigus. This requires a comprehensive judgment based on different clinical manifestations and pathological findings. Immunofluorescence and circulating autoantibody testing may be critical for some patients with atypical clinical features.

The exact mechanism of nivolumab induced SJS/TEN is not fully understood. PD-1/PD-L1 is involved in maintaining the structural and functional integrity of the skin mucosa by protecting keratinocytes (22, 23). The antagonism of PD-1 leads to the imbalance of T cell homeostasis in the skin, which leads to cytotoxic reactions and inflammatory responses. T cell overactivation can synthesize and secrete a variety of effector molecules, such as perforin/granase, FAS ligand, tumor necrosis factor. These effector molecules enter the mucosa, skin and subcutaneous tissues to activate the apoptosis process, causing the apoptosis and necrosis of keratinocytes (24, 25). Anti-pd-1/PD-L1 treatment significantly upregulate the expression of PD-L1, inflammatory chemokines (such as CXCL9, CXCL10, and CXCL11), cytotoxic factors (such as PRF1 and GZMB), and pro-apoptotic molecules FASLG in lymphocyte and keratinocytes (12).

For patients diagnosed with SJS/TEN, immediate discontinuation of the offending medication is a top priority. Effective treatment should be taken as early as possible according to the severity of the lesion, especially the area of epidermolysis. In addition to supportive treatment, systemic steroids are the primary drug of concern for STS/TEN. Systemic steroids should be given orally as 1 to 2 mg·kg^-1^·d^-1^ prednisone or methylprednisolone (26, 27). Most patients can improve their skin symptoms after receiving systemic steroids. Cyclosporin, TNF-α inhibitors, and IVIG are also used as additional treatments for poor systemic steroid response. Given the long half-life of nivolumab, plasma exchange can be an effective treatment, especially in cases where other treatments are not effective or contraindicated (28). Nivolumab induced SJS/TEN patients may have water and electrolyte disturbance, severe infection, sepsis and even septic shock. Therefore, water and electrolyte balance should be maintained during treatment, and multiple organ failure should be closely monitored and prevented. The overall mortality rate for SJS/TEN was 22%, with 4.8% for SJS, 19.4% for SJS-TEN, and 14.8% for TEN (29, 30). The overall mortality rate of nivolumab -induced SJS/TEN was 22.6%, similar to that reported. It should be noted that these seven deaths were all TEN patients. This means that the mortality rate of 13 patients with TEN is as high as 53.8%. According to our findings, the mortality rate of patients during steroid treatment alone was 7.7%, while the mortality rate during steroid combination treatment was 28.6%. However, the efficacy and safety of different treatment options still need more clinical practice to prove. Previously reported risk factors for SJS/TEN mortality include elderly patients, comorbidities, hematological cancers, sepsis, pneumonia, and renal impairment (31). In these nivolumab-induced SJS/TEN deaths, the main causes of death were infection and tumor progression. Zhou et al. believed that the degree of epidermal detachment was a key prognostic factor for ICI induced SJS/TEN (30). Three patients affected at least 80% or more of the body surface area.

### Limitation

The limitations of the study need to be discussed. First, the patients in this article were mainly from case reports, leading to reporting bias. This leads to the possibility that the findings may not be representative of all nivolumab-induced SJS/TEN patients. Second, we used two language searches, which resulted in a limited number of cases. Third, not all clinical data are reported in every article. Despite its limitations, this study is useful in providing insights into the clinical features, diagnosis, management, and outcomes of nivolumab -induced SJS/TEN. More research is needed to confirm the findings in future studies.

## Conclusion

With the widespread use of nivolumab in the cancer field, physicians should be aware of the serious skin adverse effects of nivolumab. When SJS/TEN is clinically suspected, patients should stop nivolumab immediately. Nivolumab-induced SJS/TEN is characterized by long latency, severe clinical symptoms and poor prognosis. Therefore, close monitoring, identification and treatment should be paid attention to during clinical use and after withdrawal.

## Data Availability

The original contributions presented in the study are included in the article/supplementary material. Further inquiries can be directed to the corresponding author.
